# MXene Anchored with Platinum Cobalt Alloy as an Efficient and Stable Electrocatalyst for Hydrogen Evolution

**DOI:** 10.3390/molecules29235793

**Published:** 2024-12-07

**Authors:** Jing Xiao, Buxiang Wang, Qing Shu

**Affiliations:** School of Chemistry and Chemical Engineering, Jiangxi University of Science and Technology, Ganzhou 341000, China; scorpion.xj@163.com (J.X.); wangbuxiang98@163.com (B.W.)

**Keywords:** Ti_3_C_2_T_x_, PtCo alloy, electrocatalysis, hydrogen evolution

## Abstract

It is a great challenge to prepare efficient and stable electrocatalysts for hydrogen evolution (HER) using non-precious metals. In this study, a series of PtCo/Ti_3_C_2_T_x_-Y (Y: 16, 32, and 320, Y indicates the quality of Co(NO_3_)_2_) catalysts were synthesized by loading PtCo alloy on Ti_3_C_2_T_x_. The PtCo/Ti_3_C_2_T_x_-32 catalyst showed the best HER performance, reaching a current density of 10 mA cm^−2^ with low overpotential (36 and 101 mV) and small Tafel slopes (66.37 and 105.17 mV dec^−1^) in 0.5 mol L^−1^ H_2_SO_4_ and 1 mol L^−1^ KOH solution. The excellent HER activity of PtCo/Ti_3_C_2_T_x_-32 can be attributed as follows: Ti_3_C_2_T_x_ has a unique two-dimensional structure, which can provide a large number of attachment sites for the PtCo alloy, hence exposing more active sites; on the other hand, it can also provide a fast and efficient conductive network for electron transport during electrocatalytic processes, thus enhancing the connectivity between electrolyte and catalyst. PtCo alloy makes the PtCo/Ti_3_C_2_T_x_ catalyst more hydrophilic, accelerating the release rate of bubbles. The DFT calculation results showed that the energy barrier of HER is reduced because the charge around Pt is redistributed by Co after alloying Pt and Co, so that the PtCo/Ti_3_C_2_T_x_ catalyst has a suitable ΔG_H*_ value. This study can be expected to provide some references for the design and synthesis of Ti_3_C_2_T_x_-supported alloy electrocatalysts with high activity and stability for HER.

## 1. Introduction

In the world, energy crisis and environmental pollution are becoming increasingly serious. To address the above issues, the development of clean energy conversion systems is essential to meet the growing global energy demand and achieve zero CO_2_ emissions. Hydrogen is a kind of clean energy carrier and has a broad development prospect. At present, the main green hydrogen production technology is electrochemical water hydrolysis, but it is less efficient due to the high cost [1,2]. Electrochemical water splitting includes oxygen evolution reaction (OER) and hydrogen evolution reaction (HER). The main product of HER is hydrogen, so it is an important step in electrochemical water splitting [3,4].

However, large-scale hydrogen production through HER has not yet been realized, and the key is to develop efficient, low overpotentials and low-cost electrocatalysts. The electrocatalytic activity of the hydrogen evolution electrode mainly depends on its Gibbs free energy value of hydrogen adsorption (ΔG_H*_), which is too high or too low, resulting in the difficulty of hydrogen adsorption or desorption. Since the ΔG_H*_ value of Pt-based materials is almost zero, it is currently recognized as the electrocatalyst with the best HER performance. However, due to limited reserves of Pt and its high price, Pt-based catalysts are not suitable for commercial hydrogen production [5]. Therefore, the development of efficient and low-cost HER electrocatalysts is an urgent task.

At present, the development of HER electrocatalysts based on transition metals (such as Fe, Co, Ni, etc.) has attracted wide attention [6]. Compared with the disadvantages of precious metals, such as small reserves and high market prices, transition metals have abundant reserves and cost advantages and are increasingly recognized globally. In recent years, it has been found that alloying one or two transition metals with precious metals is an effective strategy for developing efficient and stable electrocatalysts. Wang et al. synthesized a series of Ni-M (M = Ti, V, Cu, etc.) bimetallic alloys and used them as HER electrocatalysts. It was found that NiCu alloy had the best HER performance, and the current densities of 10 mA cm^−2^ and 100 mA cm^−2^ can be reached at the overpotential of 23 and 69 mV, respectively [7]. Therefore, if the transition metal is used to form an alloy with Pt, it can be expected that Pt’s utilization rate and catalytic activity of Pt can also be improved because the transition metal can regulate Pt’s charge distribution.

The introduction of a suitable support to anchor Pt-based active species is also a feasible way to improve its utilization efficiency [8]. At present, it has been reported that MXene (Ti_3_C_2_T_x_) is suitable for supporting alloys to synthesize efficient HER catalysts [9,10,11]. Ti_3_C_2_T_x_ is the general term for a series of novel two-dimensional (2D) transition metal carbides and carbon nitride materials with graphene-like structures. Wu et al. synthesized an electrocatalyst Pt/Ti_3_C_2_T_x_ with HER performance comparable to that of commercial Pt/C by anchoring sub-nanometer platinum clusters on 3D crumpled Ti_3_C_2_T_x_. It can reach a current density of 10 mA cm^−2^ at a low overpotential of 34 mV. It also has a small Tafel slope (29.7 mV dec^−1^) and a superior mass activity (1847 mA mg_Pt_^−1^). The high HER performance of Pt/Ti_3_C_2_T_x_ can be attributed to the charge transfer of Pt clusters to Ti_3_C_2_T_x_, which weakened the adsorption of hydrogen [12].

This study attempts to synthesize an electro-catalyst PtCo/Ti_3_C_2_T_x_ with high HER performance through the synergistic action between Ti_3_C_2_T_x_ (carrier) and PtCo alloy (active component). The synergistic action path is as follows: Ti_3_C_2_T_x_ are prone to stack due to the influence of van der Waals forces, which will result in the masking of catalytic active sites. However, as the PtCo alloy enters the interior of Ti_3_C_2_T_x_, it can provide structural support for Ti_3_C_2_T_x_, thus preventing the accumulation of Ti_3_C_2_T_x_. Meanwhile, Ti_3_C_2_T_x_ can enhance the interface electron/charge transfer effect between the surface of the hydrogen evolution electrode and the electrolyte. Furthermore, the structure, morphology, crystal phase, and elemental valence of the obtained catalyst were analyzed by various characterization methods, and their activity and stability during electrocatalysis of HER were studied.

## 2. Experiment

### 2.1. Reagents

Reagents: Ti_3_AlC_2_ (analytical grade, Zhejiang Yamei Nano Technology Co., Ltd., Jiaxing, China), HF (analytical grade, Shanghai Aladdin Technology Co., Ltd., Shanghai, China), Co(NO_3_)_2_·6H_2_O, H_2_PtCl_6_·6H_2_O and H_2_SO_4_, KOH were all analytically pure grade reagents and were purchased from Sinopharm Chemical Reagent Co., Ltd., Shanghai, China).

### 2.2. Catalyst Synthesis

#### 2.2.1. Ti_3_C_2_T_x_ Preparation

Ti_3_C_2_T_x_ was prepared according to the method reported in the literature [10]. The details were as follows: first, 20 mL of 48% HF aqueous solution was transferred to a 200 mL Teflon beaker. Subsequently, 1.00 g Ti_3_AlC_2_ powder was slowly added to the above-mentioned beaker within 30 min and was electromagnetically stirred for 24 h at 155 °C and 150 rpm. After that, the obtained solution was centrifuged for 10 min at 6400 rpm to obtain precipitation and supernatant (including the etched Al layers and HF solution), respectively. The resulting sediment was then repeatedly cleaned with deionized water until the pH of the dispersion was close to 6. Finally, the obtained powder was dried in a vacuum oven at 60 °C for 24 h, and the obtained powder was identified as Ti_3_C_2_T_x_.

#### 2.2.2. PtCo/Ti_3_C_2_T_x_ Preparation

**Figure 1:**
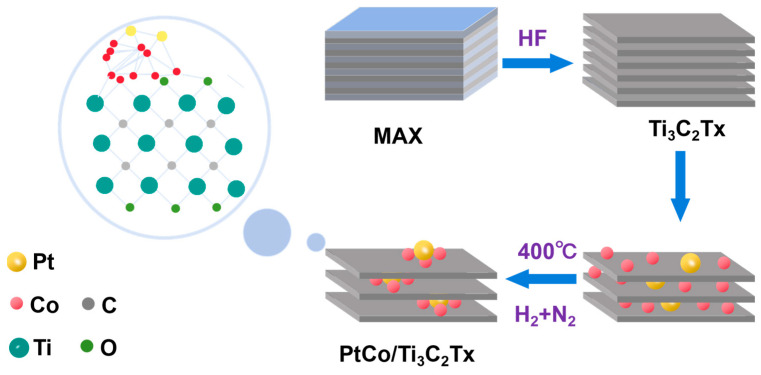
Schematic diagram of preparation of PtCo/Ti_3_C_2_T_x_.

PtCo/Ti_3_C_2_T_x_ preparation. The details are as follows: first, three parts of Ti_3_C_2_T_x_, each weighing 0.25 g, were placed in three beakers filled with 25 mL deionized water, ultrasonic treatment with an ice water bath for 2 to 3 h, and transferred to three round-bottom flasks. Second, 16.00 mg, 32.00 mg, and 320.00 mg of Co(NO_3_)_2_ were added to the above Ti_3_C_2_T_x_ solution, and electromagnetic stirring was performed at 800 rpm and 25 °C for 30 min. After standing for 3 h, the solution became clear and stratified, and the Co/Ti_3_C_2_T_x_ solution was obtained. After that, 6.64 mg H_2_PtCl_6_·6H_2_O was placed in the obtained Co/Ti_3_C_2_T_x_ solution and subjected to electromagnetic agitation for 30 min at 800 rpm and 25 °C. The precipitate and supernatant were obtained by standing in the ice bath for 12 h. The resulting sediment was dried in a vacuum oven for 8 h to obtain a black flake solid. The resulting black solid was dried in a vacuum oven at 60 °C for 8 h and then ground into powder. Finally, the powder was placed in a Muffle furnace and reduced by a mixture of 10% hydrogen and 90% nitrogen. After a 2 h reduction at 400 °C, PtCo/Ti_3_C_2_T_x_ was obtained. According to the quality of Co(NO_3_)_2_ used in the preparation of PtCo/Ti_3_C_2_T_x_ catalyst, the obtained catalysts were named PtCo/Ti_3_C_2_T_x_-16, PtCo/Ti_3_C_2_T_x_-32, and PtCo/Ti_3_C_2_T_x_-320, respectively. The synthesis route of the PtCo/Ti_3_C_2_T_x_ catalyst was shown in Figure 1.

#### 2.2.3. Pt/Ti_3_C_2_T_x_ and Co/Ti_3_C_2_T_x_ Preparation

The preparation process of Pt/Ti_3_C_2_T_x_ and Co/Ti_3_C_2_T_x_ were the same as PtCo/Ti_3_C_2_T_x_ except that Co or Pt was not added. During the preparation of Pt/Ti_3_C_2_T_x_, 3.32 mg, 6.64 mg, and 13.28 mg H_2_PtCl_6_·6H_2_O were added to the solution containing 0.3 g Ti_3_C_2_T_x_, respectively. Depending on the quality of H_2_PtCl_6_·6H_2_O, the obtained catalysts were named Pt/Ti_3_C_2_T_x_-3.32, Pt/Ti_3_C_2_T_x_-6.64, and Pt/Ti_3_C_2_T_x_-13.28, respectively. During the preparation of Co/Ti_3_C_2_T_x_, 32.00 mg of Co(NO_3_)_2_ was added to the Ti_3_C_2_T_x_ solution, and the obtained catalyst was named Co/Ti_3_C_2_T_x_-32 according to the quality of Co(NO_3_)_2_.

### 2.3. Catalyst Characterization

The surface morphologies and chemical compositions of resulting catalysts were conducted by scanning electron microscope (SEM) (MLA650F, FEI Company, Hillsboro, OR, USA) equipped with an energy dispersive X-ray spectroscopy (EDS, Quantax 400, Bruker, Ettlingen, Germany) with an accelerating voltage of 20 kV. The crystalline phases of the resulting catalyst were analyzed by X-ray diffraction (XRD) (PANalytical, Empyean, Almelo, The Netherlands) with Cu Kα (λ = 0.15418 nm) radiation under 40 kV, 40 mA over 2θ degree from 10° and 80°. The morphology of resulting catalysts was characterized by transmission electron microscope (TEM) (Tecnai G2 20, FEI, USA) with an accelerating voltage of 200 kV, point resolution of 0.25 nm and line resolution of 0.102 nm. The specific surface area and pore volume of resulting catalysts were measured by N_2_ physical adsorption instrument (ASAP 2020, Micromeritics Instrument Ltd., Norcross, GA, USA) at −196 °C. The specific surface area was calculated from the adsorption branch of the isotherm using the single-point Brunauer–Emmett–Teller (BET) method. The pore size distribution and pore volume were calculated from the desorption branch of the isotherm using the Barrett–Joyner–Halenda (BJH) method. The contact angle of the resulting catalysts was measured by a contact angle measurement system (JY-Pha, Chengde Yote Instrument Manufacturing Co. Ltd., Chengde, China) with a film thickness of 0.08 mm at intervals of 0.5 to 5 s. The elemental composition, valence state, and content of catalysts were determined by X-ray photoelectron spectroscopy (XPS) (Escalab Xi+, Thermo Fisher Scientific Inc., Waltham, MA, USA) using a hemispheric 180° dual-focus analyzer and a monochromatic Al Kα (hv = 1486.6 eV) radiation. The XPS data were analyzed using Thermo Advantage software v5.9.

### 2.4. Electrocatalytic Hydrogen Evolution Test of Catalyst

For the preparation of the test electrode, the details are as follows: first, 1 mg catalyst was weighed and ground into powder. Second, the powder was dispersed in a solution consisting of 0.25 mL isopropyl alcohol, 0.75 mL deionized water, and 20 μL 5 wt% Nafion solution. Ultrasonic dispersion in an ice water bath until a uniformly dispersed suspension was formed. Finally, the solution containing the catalyst was evenly coated on the surface of 1 cm^2^ carbon paper, and the working electrode was obtained after complete drying.

The electrocatalytic HER performance of the resulting catalyst was tested by using an electrochemical workstation (CHI660E, Shanghai Chenhua Instrument Co., Ltd., Shanghai, China) at 25 °C. A standard three-electrode system was adopted, and the resulting catalyst, graphite rod, and saturated calomel electrode (SCE) served as the working electrode, counter electrode, and reference electrode, respectively. All the potentials were converted to the reversible hydrogen electrode (RHE) after the measurements using Equation (1):

(1) $$V_{\left(RHE\right)}=V_{\left(SCE\right)}+0.0591\times pH+0.2415$$

where V_(RHE)_ is the potential value (V) of RHE, V_(SCE)_ is the potential value (V) of SCE, and pH is the pH value of the solution.

Cyclic voltammetry (CV) was used to measure the charging and discharging current of the electric double layer of the resulting catalyst in the potential range without electrochemical reaction. In the linear relationship between the non-Faraday current of the resulting catalyst and the linear scanning rate, the slope is the electrochemical double-layer capacitance (C_dl_) of the catalyst sample, as shown in Equation (2).

(2) $$I=V\times C_{dl}$$

where V is the scanning rate (mV s^−1^), and I is the current density (mA cm^−2^) at different scanning rates.

In general, the electro-catalytic HER activity of different catalysts can be compared in terms of the overpotential (η) values required to achieve a 10 mA cm^−2^ current density. In principle, η values can be obtained by the linear sweep voltammetry (LSV) curve. In this study, LSV curves were obtained at a low scan rate of 5 mV s^−l^. Then, we made a straight line y = −10 on the LSV curve, and the abscissa of the intersection point with the LSV curve was the η value after taking the absolute value.

The Tafel slope is the rate at which the current increases with respect to η, which is mainly determined by the transfer coefficient. It can be obtained from the Tafel diagram derived from the LSV result and then fitting the linear portion at the lower η by the Tafel equation as shown in Equation (3).

(3) η=blogI+a

where η is the overpotential (mV), I is the current density (mA cm^−2^), a is the constant, and b is the Tafel slope.

The mass activity (MA) reflects the true electrocatalytic activity of the electrocatalyst. In order to more intuitively compare the activity of electrocatalytic different electrocatalysts, MA values of them were calculated according to Equation (4):

(4) MA=I/M

where MA (mA mg^−1^) is the mass activity of the catalyst, I is the current density (mA cm^−2^), and M is the catalyst load (mg cm^−2^).

The stability of the resulting electrocatalyst was tested by the time-current (i-t) method which was performed in 1 mol L^−1^ KOH and 0.5 mol L^−1^ H_2_SO_4_ electrolyte solutions, and the obtained data were not compensated by IR. The measurement of i-t was conducted under a constant applied potential of 0.8 V (vs. RHE).

### 2.5. DFT Calculation

The density functional theory (DFT) was used to calculate the state density of PtCo/Ti_3_C_2_T_x_ by the plane wave method in the Vienna Ab initio Simulation Package v6.1 (VASP). The exchange correlation was approximated within the generalized gradient approximation (GGA) in the Dmol^3^ module with the Perdew–Burke–Ernzerhof (PBE) method. The interaction between ionic cores and valence electrons was described by the ultrasoft pseudopotential (USPP) method. The pseudopotential valences of Pt and Co were [Xe]4f^14^5d^9^6s^1^ and [Ar]3d^7^4s^2^, respectively. The Monkhorst–Pack k-point mesh of 2 × 2 × 1 was adopted, and the cut-off energy for plane wave expansion was set to 500 eV and the electronic self-consistency was 10^−4^ eV atom^−1^. The vacuum layer thickness in the z direction is set to 15 A, which makes the interaction between the two adjacent catalyst surfaces negligible. Here, ΔG_H*_ on different catalyst surfaces can be calculated by Equation (5).

(5) $$\Delta G_{H*}=G_{tot}-G_{slab}-\frac{1}{2}G_{H_{2}}(g)$$

where G_tot_ and G_slab_ represent the total energy of the catalyst system with and without adsorbed hydrogen atoms on the surface, respectively, and $G_{H_{2}}(g)$ represents the total energy of a gas phase H_2_ molecule.

## 3. Results and Discussion

### 3.1. Characterization of PtCo/Ti_3_C_2_T_x_ Catalyst

**Figure 2:**
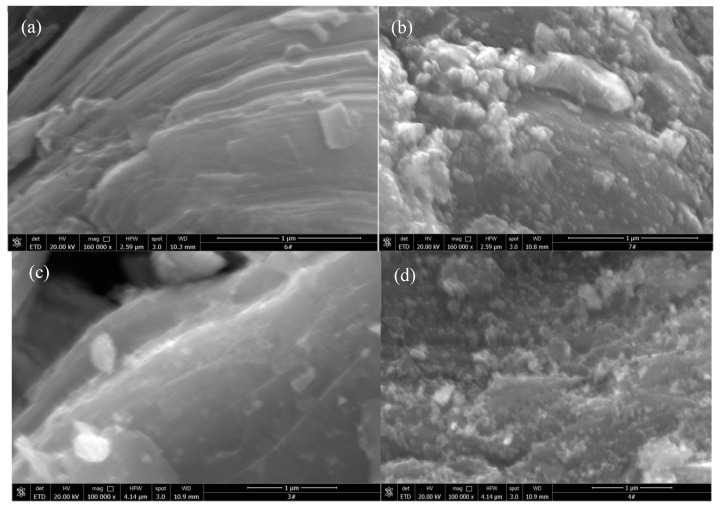
SEM images of (**a**) Ti_3_C_2_T_x_, (**b**) Pt/Ti_3_C_2_T_x_-6.64, (**c**) Co/Ti_3_C_2_T_x_-32, (**d**) PtCo/Ti_3_C_2_T_x_-32.

The morphology of Ti_3_C_2_T_x_, Pt/Ti_3_C_2_T_x_-6.64, Co/Ti_3_C_2_T_x_-32, and PtCo/Ti_3_C_2_T_x_-32 catalysts were analyzed by SEM, and the results were shown in Figure 2. As can be seen from the SEM images of Ti_3_C_2_T_x_ shown in Figure 2a, Ti_3_C_2_T_x_ presents a multi-layer and compact stack lamellar structure, which is a typical two-dimensional layered structure. The unique two-dimensional structure of Ti_3_C_2_T_x_ can provide an effective conductive network for fast ion and electron transport during the electrocatalysis HER process [13]. From the SEM image of Pt/Ti_3_C_2_T_x_-6.64 shown in Figure 2b, it can be clearly seen that granular Pt particles were deposited on the surface of Ti_3_C_2_T_x_. As can be seen from the SEM image of Co/Ti_3_C_2_T_x_-32 shown in Figure 2c, the morphology of Ti_3_C_2_T_x_ has not changed significantly. It still presents a two-dimensional layered structure, and no obvious granular particles were found on its surface. From the SEM image of PtCo/Ti_3_C_2_T_x_-32 shown in Figure 2d, it can be seen that the particle size loaded on the surface of PtCo/Ti_3_C_2_T_x_-32 was significantly smaller than that of Pt/Ti_3_C_2_T_x_-6.64, indicating that Co can effectively inhibit the agglomeration of Pt.

In order to understand the type and distribution of elements in the PtCo/Ti_3_C_2_T_x_-32 catalyst as shown in Figure S1a (Supporting Information), EDS elemental mapping analysis was conducted, and analysis results were shown in Figure S1b–f (Supporting Information). It can be found that there are many bright regions with a uniform distribution of particles, which indicates that Pt and Co did not form large particles on the surface of Ti_3_C_2_T_x_. Combined with the SEM analysis results, it can be found that Co not only inhibits the aggregation of Pt to form large-sized particles, but also promotes the uniform distribution of Pt on the surface of Ti_3_C_2_T_x_, which is beneficial to the exposure of more active sites of the catalyst [14]. Element point scanning analysis was carried out on PtCo/Ti_3_C_2_T_x_-32, that is, six points were randomly selected on its surface to detect the element information of each point, and the results were shown in Figure S1g (Supporting Information). As can be seen from the results of Figure S1g, the distribution of Pt and Co elements was relatively uniform. The average atomic percentages of C, Ti, Co and Pt were 37.67, 61.23, 1.03 and 0.07, respectively. It can be inferred that the mass percentage of C, Ti, Co and Pt were 13.04, 84.81, 1.75 and 0.39, respectively.

**Figure 3:**
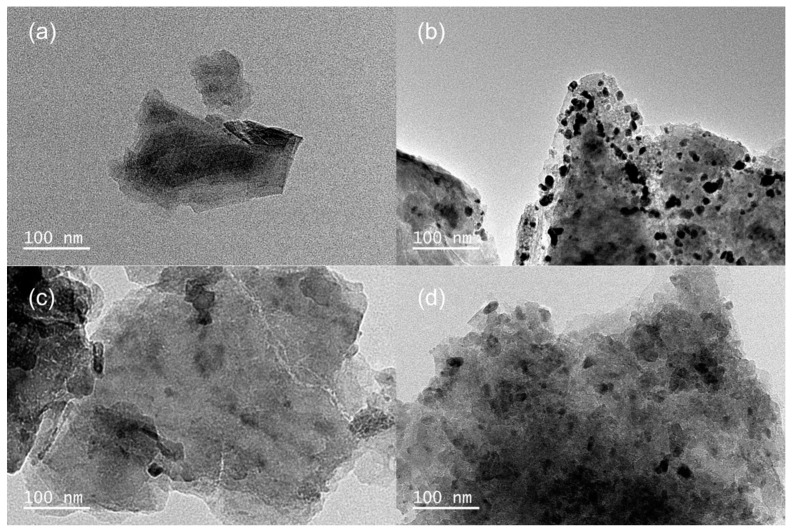
TEM images of (**a**) Ti_3_C_2_T_x_, (**b**) Pt/Ti_3_C_2_T_x_-6.64, (**c**) Co/Ti_3_C_2_T_x_-32, (**d**) PtCo/Ti_3_C_2_T_x_-32.

In order to further study the microstructure of the resulting catalysts, the structures of Ti_3_C_2_T_x_, Pt/Ti_3_C_2_T_x_-6.64, Co/Ti_3_C_2_T_x_-32 and PtCo/Ti_3_C_2_T_x_-32 were analyzed by TEM, and the results were shown in Figure 3. It can be seen from Figure 3a that Ti_3_C_2_T_x_ has a unique two-dimensional layered structure. It can be found from Figure 3b that a large number of clusters with different sizes were attached to flaky Ti_3_C_2_T_x_. This is because Pt particles are prone to agglomerate during the preparation process, which may seriously affect the activity and stability of the catalyst [15]. Figure 3c showed the TEM image of Co/Ti_3_C_2_T_x_-32, and no obvious agglomeration phenomenon was found, which is consistent with the SEM analysis results. From the TEM image of PtCo/Ti_3_C_2_T_x_-32 shown in Figure 3d, it can be seen that the number of clusters in PtCo/Ti_3_C_2_T_x_-32 is significantly reduced and the size is smaller than that of Pt/Ti_3_C_2_T_x_. It can be concluded that Co can inhibit the formation of large Pt particles, which will help to expose more Pt to the catalyst surface, thereby improving the utilization rate of Pt.

**Figure 4:**
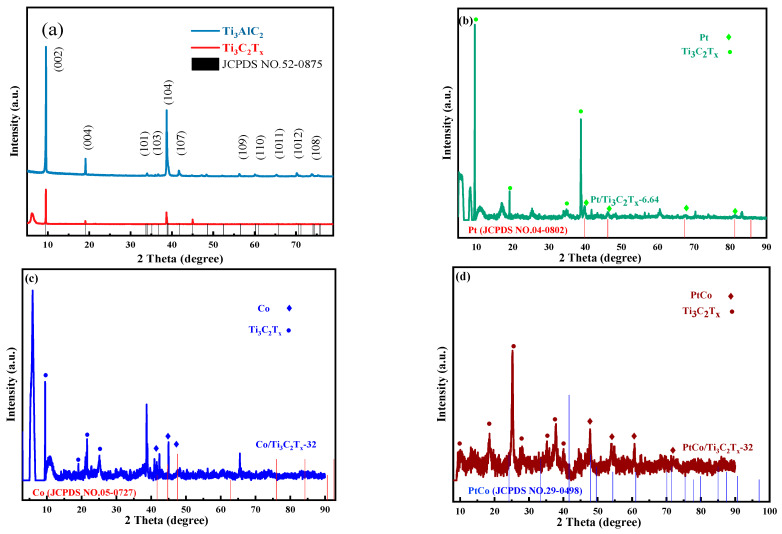
XRD patterns of (**a**) Ti_3_AlC_2_ and Ti_3_C_2_T_x_, (**b**) Pt/Ti_3_C_2_T_x_-6.64, (**c**) Co/Ti_3_C_2_T_x_-32, and (**d**) PtCo/Ti_3_C_2_T_x_-32.

The phase structures of Ti_3_AlC_2_, Ti_3_C_2_T_x_ and Pt/Ti_3_C_2_T_x_-6.64, Co/Ti_3_C_2_T_x_-32, and PtCo/Ti_3_C_2_T_x_-32 were analyzed by XRD, and the results were shown in Figure 4. Figure 4a showed the XRD analysis results of Ti_3_AlC_2_ and Ti_3_C_2_T_x_. It can be seen from Figure 4a that after HF etching of Ti_3_AlC_2_, three distinct characteristic diffraction peaks appeared at 2θ of 9.5°, 19.3°, and 39.0°. It can be known that the above characteristic diffraction peaks, respectively, correspond to (002), (004), and (104) crystal faces after comparing the Ti_3_AlC_2_ standard card (JCPDS NO. 52-0875). In addition, a new broad characteristic diffraction peak appeared at 2θ of 6.1°, indicating that the Al layer has been etched and that Ti_3_AlC_2_ has successfully transformed into Ti_3_C_2_T_x_. It also indicated that the layer spacing of Ti_3_C_2_T_x_ has become larger after the etching of the Al layer, which facilitates the entry of active substances into Ti_3_C_2_T_x_ [9]. It can be seen from Figure 4b that Co/Ti_3_C_2_T_x_-32 appeared at three obvious characteristic diffraction peaks at 2θ of 41.6°, 44.9°, and 47.5°, respectively. These characteristic peaks are indexed to the hcp Co phase (JCPDS 05-0727), corresponding to the crystal faces of Co (100), (002), and (101), respectively. In addition, Co/Ti_3_C_2_T_x_-32 also exhibited two characteristic diffraction peaks at 2θ of 41.0° and 42.4°, and these above two characteristic diffraction peaks were indexed to the (321) crystal face of CoTi_2_O_5_ and the (200) crystal face of CoO after comparing the standard card of CoTi_2_O_5_ (JCPDS 35-0793) and CoO (JCPDS NO. 43-1004), respectively. It can also be found that the intensity of the characteristic diffraction peaks at 2θ of 41.0° and 42.4° corresponding to the CoTi_2_O_5_ (321) crystal plane and CoO (200) crystal plane was very weak, indicating that Co/Ti_3_C_2_T_x_-32 contains only a small amount of CoTi_2_O_5_ and CoO. The Co element mainly exists in the form of Co. It can be seen from Figure 4c that Pt/Ti_3_C_2_T_x_ appeared two characteristic diffraction peaks at 2θ of 39.7° and 46.2°, and these characteristic peaks were indexed to the (111) and (200) crystal faces of Pt after comparing the Pt standard card (JCPDS NO. 04-0802). Moreover, Co/Ti_3_C_2_T_x_-32 and Pt/Ti_3_C_2_T_x_-6.64 show the same diffraction peak at 2θ of 41.7°, corresponding to the Ti_3_C_2_T_x_ substrate, which indicates that Co and Pt have successfully loaded on Ti_3_C_2_T_x_. By observing the XRD pattern of PtCo/Ti_3_C_2_T_x_ as shown in Figure 4d, four characteristic diffraction peaks appeared at 2θ of 47.9, 54.4, 61.1, and 71.4 corresponding to the PtCo alloy after comparing the standard card of PtCo alloy (JCPDS NO. 29-0498). It is worth noting that the XRD pattern of PtCo/Ti_3_C_2_T_x_ does not show any diffraction peaks corresponding to the atomic and oxidation states of Co(Pt). The results show that Co and Pt exist in the form of PtCo alloy in PtCo/Ti_3_C_2_T_x_.

**Figure 5:**
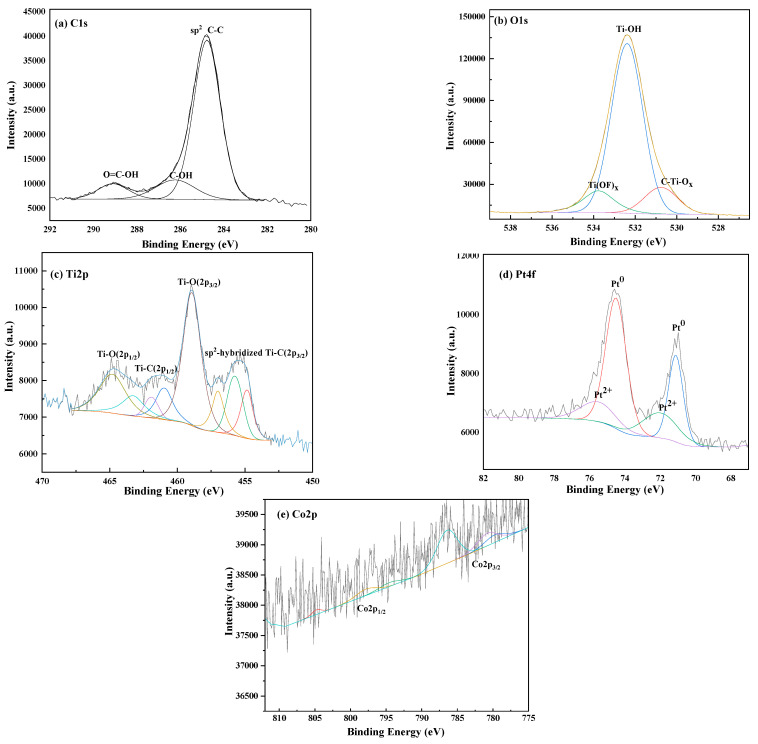
XPS diagram of PtCo/Ti_3_C_2_T_x_-32.

The chemical states and valence electron structures of the surface atoms of Ti_3_C_2_T_x_, Co/Ti_3_C_2_T_x_-32, Pt/Ti_3_C_2_T_x_-6.64, and PtCo/Ti_3_C_2_T_x_-32 electrocatalysts were studied by XPS. From Figure S2a to Figure S4a (Supporting Information) and Figure 5a showed the spectrum of C1s of these above electrocatalysts (Supporting Information). The peak at 284.8 eV corresponds to C-C bond, and the other three peaks correspond to the sp^2^ C-C (284.7 eV), C-OH (286.2 eV), and O=C-OH (288.9 eV), respectively [16,17]. Figure S2b to Figure S4b (Supporting Information) and Figure 5b showed the spectrum of O1s of these above electrocatalysts, three peaks at 530.3 eV, 531.8 eV, and 533.6 eV correspond to the C-Ti-O_x_ bond, Ti-OH bond and Ti(OF)_x_, respectively [18]. Figure S2c to Figure S4c (Supporting Information) and Figure 5c showed the Ti2p spectra of these above electrocatalysts, and the characteristic peaks at the binding energies of 455.3, 459.1, 461.4, and 464.9 eV was ascribed to sp2-hybridized Ti-C(2p_3/2_), Ti-O(2p_3/2_), Ti-C(2p_1/2_), and Ti-O(2p_1/2_) groups, respectively [19]. The presence of TiO_2_ component in the catalyst should be attributed to the inevitable surface oxidation of the Ti_3_C_2_T_x_ in the air. It has been proven to be unavoidable and non-influence on the structure and physicochemical properties of Ti_3_C_2_T_x_ [20]. Figure S4d (Supporting Information) and Figure 5d shows the Pt4f spectra of Pt/Ti_3_C_2_T_x_-6.64 and PtCo/Ti_3_C_2_T_x_-32. Generally, the peaks at 71.4 eV (Pt4f_7/2_) and 74.7 eV (Pt4f_5/2_) correspond to Pt^0^, and the peaks at 72.5 eV (Pt4f_7/2_) and 75.9 eV (Pt4f_5/2_) correspond to Pt^2+^, respectively [17]. It can be seen from the XPS spectra of PtCo/Ti_3_C_2_T_x_-32, the peaks corresponding to Pt^0^ and Pt^2+^ all move in the direction of lower binding energies, and the shift amplitude of Pt^2+^ was larger. Figure S3d (Supporting Information) and Figure 5e showed the Co2p spectra of Co/Ti_3_C_2_T_x_-32 and PtCo/Ti_3_C_2_T_x_-32. In the Co2p of Co/Ti_3_C_2_T_x_-32, the three characteristic peaks (779.5 eV, 781.3 eV, and 785.9 eV) in the Co2p_3/2_ region, respectively, corresponding to Co^0^, Co^3+^, and Co^2+^, indicating that Co exists in the form of metallic Co and in the oxidation state as Co_3_O_4_, which is consistent with the XRD analysis results. And more, the peak corresponding to Co 2p_1/2_ and two satellite peaks were located at 795.5 eV, 797.0, and 803.2 eV [21]. In the XPS spectra of PtCo/Ti_3_C_2_T_x_-32, peaks correspond to Co^2+^ and Co^3+^ move slightly in the direction of higher binding energies, and the peak corresponds to Pt4f moves in the direction of lower binding energies. The shift in peak position indicated that the electron distribution of Pt and Co elements has changed, that is, there is a strong electron interaction between them. This is because electrons were transferred from less electronegative Co to more electronegative Pt, resulting in a change in the electronic environment of Pt. It will affect the adsorption of the catalyst to the reactant molecules and the desorption ability of the product [22].

The surface hydrophilicity of the catalyst was determined by measuring the contact angles of Ti_3_C_2_T_x_, Pt/Ti_3_C_2_T_x_-6.64, Co/Ti_3_C_2_T_x_-32, and PtCo/Ti_3_C_2_T_x_-32 (as shown in Figure S5, Supporting Information). The contact angles of Ti_3_C_2_T_x_, Pt/Ti_3_C_2_T_x_-6.64, Co/Ti_3_C_2_T_x_-32, and PtCo/Ti_3_C_2_T_x_-32 are 130.4°, 98.1°, 52.7°, and 12.1°, respectively. The contact angle of PtCo/Ti_3_C_2_T_x_ is much smaller than that of Ti_3_C_2_T_x_, Pt/Ti_3_C_2_T_x_-6.64, and Co/Ti_3_C_2_T_x_-32, which indicates that the PtCo/Ti_3_C_2_T_x_-32 catalyst has strong hydrophilicity. The aggregation between Ti_3_C_2_T_x_ layers can be effectively inhibited after the inserting of Co particles or Pt particles into the gaps between Ti_3_C_2_T_x_ layers, thus enhancing the hydrophilicity of Ti_3_C_2_T_x_. Compared with Pt/Ti_3_C_2_T_x_, some Co on the surface of Co/Ti_3_C_2_T_x_ was oxidized to Co_3_O_4_, which further enhanced the hydrophilicity of the Ti_3_C_2_T_x_. Therefore, the hydrophilicity of Co/Ti_3_C_2_T_x_ is higher than that of Pt/Ti_3_C_2_T_x_. Because Co can inhibit the aggregation of Pt, so PtCo alloy can inhibit the aggregation of T Ti_3_C_2_T_x_ layer more effectively, and thus has the highest hydrophilicity. Good hydrophilicity can make the contact between the active site and the electrolyte more adequate, which is also conducive to improving HER reaction activity [23].

**Figure 6:**
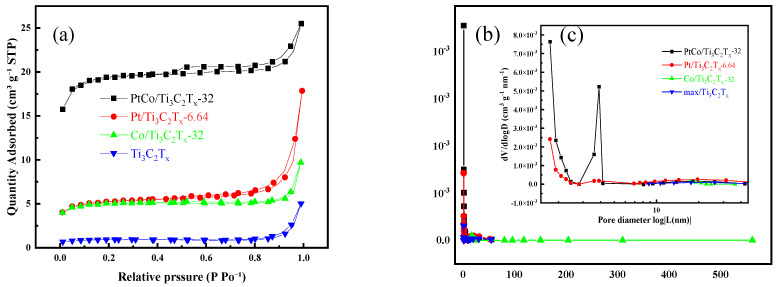
Ti_3_C_2_T_x_, Co/Ti_3_C_2_T_x_-32, Pt/Ti_3_C_2_T_x_-6.64, and PtCo/Ti_3_C_2_T_x_-32 of (**a**) N_2_ adsorption–desorption curve, (**b**) Pore size distribution, illustration, (**c**) Pore size distribution (0–50 nm).

The specific surface area and pore size distribution of Ti_3_C_2_T_x_, Pt/Ti_3_C_2_T_x_-6.64, Co/Ti_3_C_2_T_x_-32, and PtCo/Ti_3_C_2_T_x_-32 were determined by the specific surface area and pore size analyzer. Figure 6a shows the N_2_ adsorption–desorption curves of Ti_3_C_2_T_x_, Pt/Ti_3_C_2_T_x_-6.64, Co/Ti_3_C_2_T_x_-32, and PtCo/Ti_3_C_2_T_x_-32. Figure 6b shows the pore size distribution of Ti_3_C_2_T_x_, Pt/Ti_3_C_2_T_x_-6.64, Co/Ti_3_C_2_T_x_-32, and PtCo/Ti_3_C_2_T_x_-32. It can be found that PtCo/Ti_3_C_2_T_x_-32 has a wider aperture distribution when it was compared with Ti_3_C_2_T_x_ and Co/Ti_3_C_2_T_x_-32. From the pore size distribution (0–50 nm) shown in Figure 6c, it can be found that the probability of mesoporous distribution of PtCo/Ti_3_C_2_T_x_-32 was significantly higher than that of the other three catalysts, and the pore distribution was mainly between 2 and 3 nm. The N_2_ adsorption–desorption curves of Ti_3_C_2_T_x_, Pt/Ti_3_C_2_T_x_-6.64, Co/Ti_3_C_2_T_x_-32, and PtCo/Ti_3_C_2_T_x_-32 were analyzed and calculated to obtain the specific surface area, pore size, and pore volume of the above-mentioned catalysts. The results were shown in Table 1.

As can be seen from Table 1, Ti_3_C_2_T_x_ has a specific surface area of 2.96 m^2^/g, a pore volume of 0.0075 cm^3^/g, and a pore size of 10.77 nm. After loading Co with Ti_3_C_2_T_x_, the specific surface area, pore volume, and pore diameter of Co/Ti_3_C_2_T_x_-32 become 15.41 m^2^/g, 0.015 cm^3^/g, and 38.84 nm, respectively. It can be found that all the specific surface areas, pore diameters, and pore volumes increased. After loading Pt with Ti_3_C_2_T_x_, the specific surface area of Pt/Ti_3_C_2_T_x_-6.64 was 16.37 m^2^/g and the pore volume was 0.028 cm^3^/g, both the specific surface area and pore volume increased. However, the pore size was reduced to 6.75 nm. The specific surface area of PtCo/Ti_3_C_2_T_x_-32 was 59.31 m^2^/g, which was greatly improved. The pore volume was 0.039 cm^3^/g, so the pore volume also increased. The pore size is 2.66 nm, which is reduced. The results show that the specific surface area and pore volume of Pt/Ti_3_C_2_T_x_-6.64, Co/Ti_3_C_2_T_x_-32, and PtCo/Ti_3_C_2_T_x_-32 were increased when they were compared with Ti_3_C_2_T_x_, which is due to the effect of inserting Pt, Co, and PtCo particles between the Ti_3_C_2_T_x_ layers. The accumulation of Ti_3_C_2_T_x_ flakes was effectively inhibited and the layer spacing was expanded [24]. It is worth noting that the number of active sites on the catalyst surface is positively correlated with the catalytic performance of HER [25]. Therefore, PtCo/Ti_3_C_2_T_x_-32 can be expected to have the highest HER electrocatalytic activity.

### 3.2. Electrocatalytic HER Performance Test in Acidic Electrolyte

**Figure 7:**
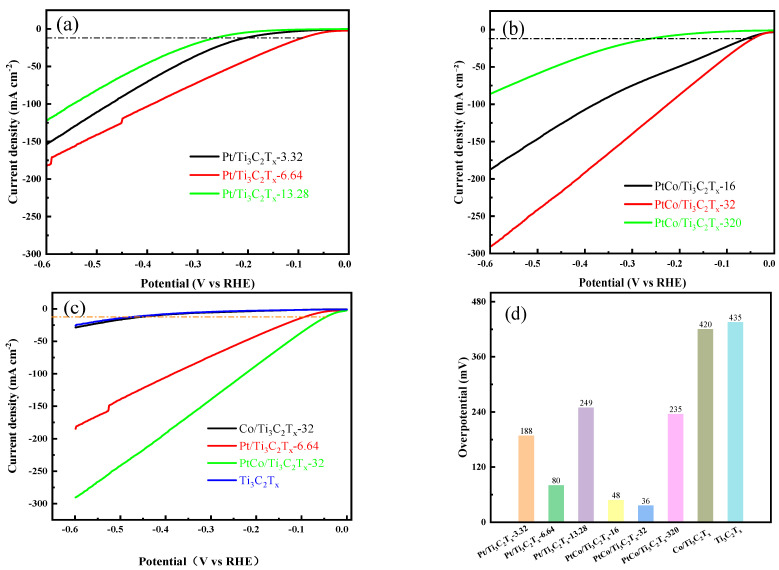
HER performance diagram of catalyst in 0.5 mol/L H_2_SO_4_ solution. LSV diagram of (**a**) Pt/Ti_3_C_2_T_x_ with different amounts of Pt, (**b**) PtCo/Ti_3_C_2_T_x_ with different amounts of Co, (**c**) Ti_3_C_2_T_x_, Co/Ti_3_C_2_T_x_-32, Pt/Ti_3_C_2_T_x_-6.64, and PtCo/Ti_3_C_2_T_x_-32; (**d**) overpotential values of catalysts.

The HER electrocatalytic performance of Pt/Ti_3_C_2_T_x_-3.32, Pt/Ti_3_C_2_T_x_-6.64, and Pt/Ti_3_C_2_T_x_-13.28 catalysts was tested in 0.5 mol/L H_2_SO_4_ solution. LSV results were shown in Figure 7a. It can be seen that the Pt/Ti_3_C_2_T_x_-6.64 catalyst has the best HER electrocatalytic performance. The possible reasons for the low catalytic activity of Pt/Ti_3_C_2_T_x_-3.32 and Pt/Ti_3_C_2_T_x_-13.28 were as follows: when the content of Pt was insufficient, there are not enough active sites on the surface of Ti_3_C_2_T_x_, resulting in poor HER performance; when the content of Pt was high, it is easier to agglomerate to form particles at high-temperature reduction, making the Pt atoms wrapped in the particles unable to participate in the reaction, resulting in insufficient active sites. Therefore, the content of Pt was fixed at 6.64 mg, and the effects of different amounts of Co on the electrocatalytic HER performance were studied. The electrocatalytic HER performance of PtCo/Ti_3_C_2_T_x_-16, PtCo/Ti_3_C_2_T_x_-32, and PtCo/Ti_3_C_2_T_x_-320 were tested in 0.5 mol/L H_2_SO_4_ solution and the LSV results were shown in Figure 7b. As can be seen from Figure 7b, the electrocatalytic HER performance of these above catalysts showed a trend of first increasing and then decreasing with the increase in Co content, and the PtCo/Ti_3_C_2_T_x_-32 catalyst exhibited the best electrocatalytic HER performance. As a comparison, the electrocatalytic HER performance of Ti_3_C_2_T_x_, Co/Ti_3_C_2_T_x_-32, Pt/Ti_3_C_2_T_x_-6.64, and PtCo/Ti_3_C_2_T_x_-32 were tested and the LSV results of these above catalysts were shown in Figure 7c. It can be seen from Figure 7c that PtCo/Ti_3_C_2_T_x_-32 illustrates the best electrocatalytic HER performance. Finally, the η values of Ti_3_C_2_T_x_, Co/Ti_3_C_2_T_x_-32, Pt/Ti_3_C_2_T_x_-6.64, and PtCo/Ti_3_C_2_T_x_-32 required to reach a current density of 10 mA cm^−2^ were obtained, as shown in Figure 7d. It can be seen from Figure 7d that the η values of Ti_3_C_2_T_x_ and Co/Ti_3_C_2_T_x_-32 were 435 mV and 420 mV, respectively. However, the η values of Pt/Ti_3_C_2_T_x_-6.64 and PtCo/Ti_3_C_2_T_x_-32 were 80 mV and 36 mV, respectively. It is worth noting that the η of commercial Pt/C catalyst was 45 mV to reach a current density of 10 mA cm^−2^ in 0.5 M H_2_SO_4_ [26], so the electrocatalytic HER performance of PtCo/Ti_3_C_2_T_x_-32 is even better than the commercial Pt/C catalyst.

**Figure 8:**
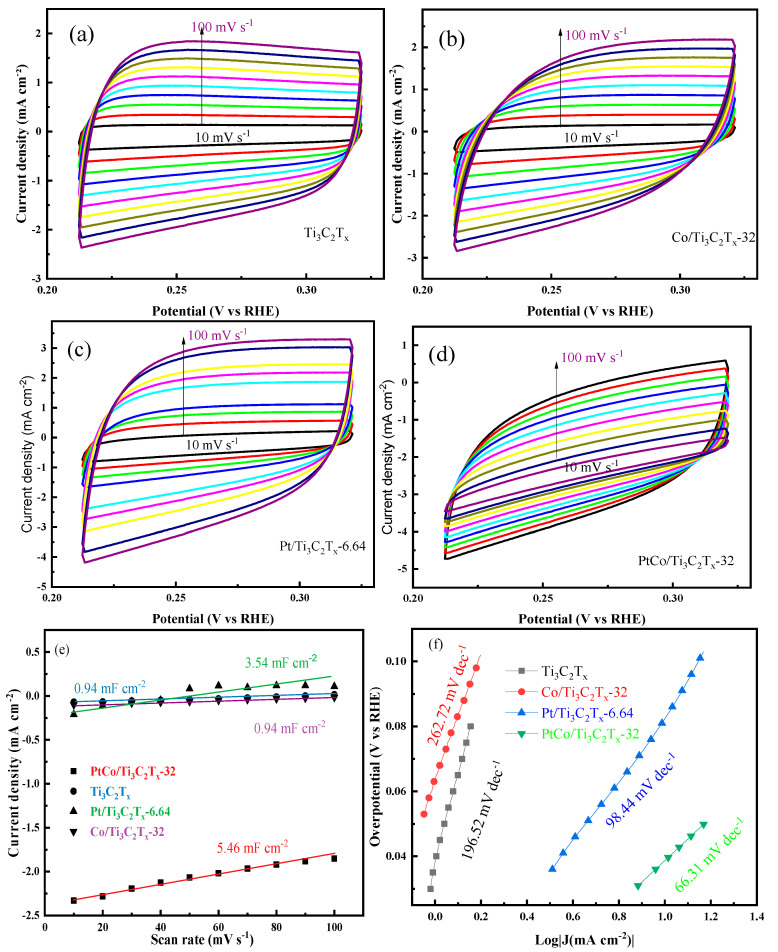
CV, C_dl_, and Tafel diagrams of Ti_3_C_2_T_x_, Co/Ti_3_C_2_T_x_-32, Pt/Ti_3_C_2_T_x_-6.64, and PtCo/Ti_3_C_2_T_x_-32 in 0.5 mol L^−1^ H_2_SO_4_ solution. CV diagram of (**a**) Ti_3_C_2_T_x_, (**b**) Co/Ti_3_C_2_T_x_-32, (**c**) Pt/Ti_3_C_2_T_x_-6.64, (**d**) PtCo/Ti_3_C_2_T_x_-32, (**e**) C_dl_ diagram, (**f**) Tafel diagrams.

In order to compare the electrocatalytic HER performance of Ti_3_C_2_T_x_, Co/Ti_3_C_2_T_x_-32, Pt/Ti_3_C_2_T_x_-6.64, and PtCo/Ti_3_C_2_T_x_-32 catalysts, the CV curves of these above catalysts at different scanning speeds were obtained and shown as Figure 8a–d. Furthermore, the C_dl_ values of these above catalysts were calculated by Equation (2) and shown as Figure 8e. It can be seen from Figure 8e that the C_dl_ values of Ti_3_C_2_T_x_, Co/Ti_3_C_2_T_x_-32, Pt/Ti_3_C_2_T_x_-6.64, and PtCo/Ti_3_C_2_T_x_-32 catalysts were 0.94 mF cm^−2^, 0.94 mF cm^−2^, 3.54 mF cm^−2^, and 5.46 mF cm^−2^, respectively. In order to further explore the HER kinetics of these catalysts, the corresponding Tafel slope values were obtained through the LSV curves of each catalyst, and the results were shown in Figure 8f. It can be seen from Figure 8f that the Tafel slope of the PtCo/Ti_3_C_2_T_x_-32 catalyst was 66.31 mV dec^−1^, which indicates that the Volmer–Heyrovsky step is rate limiting. The Tafel slopes of Pt/Ti_3_C_2_T_x_-6.64, Co/Ti_3_C_2_T_x_-32, and Ti_3_C_2_T_x_ were 98.44 mV dec^−1^, 196.52 mV dec^−1^, and 262.72 mV dec^−1^, respectively. Compared with Pt/Ti_3_C_2_T_x_-6.64, Co/Ti_3_C_2_T_x_-32, and Ti_3_C_2_T_x_, the Tafel slope of PtCo/Ti_3_C_2_T_x_-32 was significantly reduced, which means that its kinetic process is faster, indicating that the catalyst can reach the required current density at a lower η value, that is, it has the best electrocatalytic HER performance.

**Figure 9:**
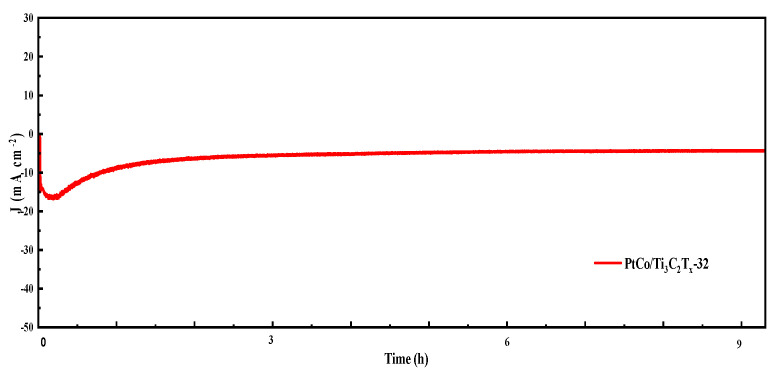
i-t test diagram of PtCo/Ti_3_C_2_T_x_-32 in 0.5 mol L^−1^ H_2_SO_4_ solution.

The electrocatalytic HER stability of PtCo/Ti_3_C_2_T_x_-32 was tested in 0.5 mol L^−1^ H_2_SO_4_ solution, and the i-t curve was shown in Figure 9.

As can be seen from Figure 9, PtCo/Ti_3_C_2_T_x_-32 showed good stability after the 10 h test. Therefore, PtCo alloying can improve the corrosion resistance of Co in an acidic media and protect the active site of the catalyst.

### 3.3. Electrocatalytic HER Performance Test in Alkaline Electrolyte

**Figure 10:**
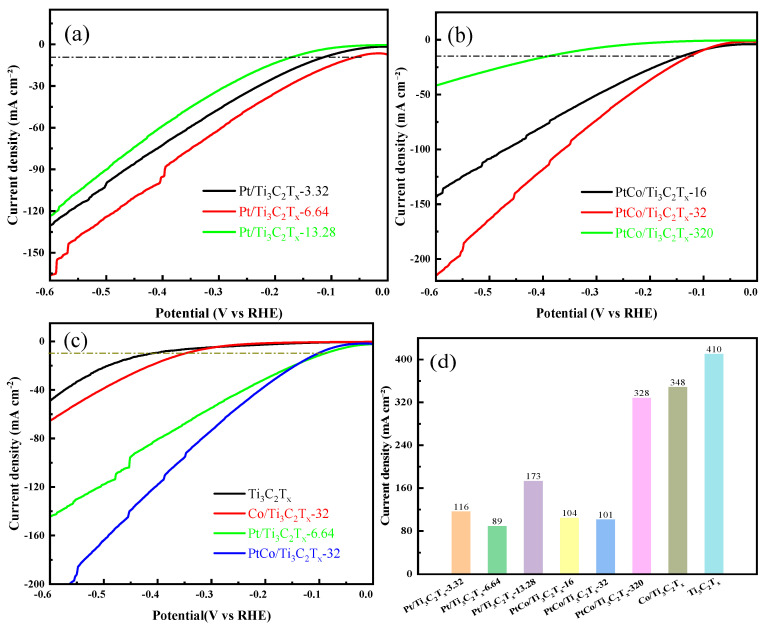
LSV diagram of (**a**) Pt/Ti_3_C_2_T_x_ with different Pt content, (**b**) PtCo/Ti_3_C_2_T_x_ with different Co content, (**c**) Ti_3_C_2_T_x_, Co/Ti_3_C_2_T_x_-32, Pt/Ti_3_C_2_T_x_-6.64, and PtCo/Ti_3_C_2_T_x_-32; and (**d**) overpotential values of catalysts.

The electrocatalytic HER performance of Pt/Ti_3_C_2_T_x_-3.32, Pt/Ti_3_C_2_T_x_-6.64, and Pt/Ti_3_C_2_T_x_-13.28 were tested in 1 mol L^−1^ KOH solution using a standard three-electrode system. It can be seen from Figure 10a that the Pt/Ti_3_C_2_T_x_-6.64 catalyst exhibited the best electrocatalytic HER performance. Subsequently, the Pt content was fixed at 6.64 mg, and the electrocatalytic HER performance of PtCo/Ti_3_C_2_T_x_-16, PtCo/Ti_3_C_2_T_x_-32, and PtCo/Ti_3_C_2_T_x_-320 was tested to understand the influence of the amount of Co. It can be seen from Figure 10b that the electrocatalytic HER performance increases first and then decreases with the increase in Co content, and PtCo/Ti_3_C_2_T_x_-32 has the best electrocatalytic HER performance. Figure 10c shows the LSV curves of Ti_3_C_2_T_x_, Co/Ti_3_C_2_T_x_-32, Pt/Ti_3_C_2_T_x_-6.64, and PtCo/Ti_3_C_2_T_x_-32. Figure 10d showed that η values of Ti_3_C_2_T_x_ and Co/Ti_3_C_2_T_x_-32 were, respectively, 410 mV and 348 mV to reach a current density of 10 mA cm^−2^, which indicated that their electrocatalytic HER performance was poor. Although the η values of Ti_3_C_2_T_x_ and Co/Ti_3_C_2_T_x_-32 in 1 mol L^−1^ KOH solution are lower than their η values in 0.5 mol L^−1^ H_2_SO_4_ solution, the overall η values were still too large. The addition of Pt improved the electrocatalytic HER performance of Pt/Ti_3_C_2_T_x_-6.64 (89 mV) and PtCo/Ti_3_C_2_T_x_-32 (101 mV). It is worth noting that the η of commercial 20% Pt/C was 304 mV to reach a current density of 10 mA cm^−2^ in 1 M KOH [27], so the electrocatalytic HER performance of PtCo/Ti_3_C_2_T_x_-32 was also better than the commercial Pt/C catalyst.

It is worth noting that the electrocatalytic HER performance of PtCo/Ti_3_C_2_T_x_-32 was inferior to that of Pt/Ti_3_C_2_T_x_-6.64 under low potential. However, the electrocatalytic HER performance of PtCo/Ti_3_C_2_T_x_-32 rapidly exceeds that of Pt/Ti_3_C_2_T_x_-6.64 with the increase in potential. In addition, the η value of the PtCo/Ti_3_C_2_T_x_-32 in 0.5 mol L^−1^ H_2_SO_4_ solution is 36 mV, and the η value becomes 101 mV after the electrolyte is replaced with 1 mol L^−1^ KOH. The results showed that PtCo/Ti_3_C_2_T_x_-32 can be used as an electrocatalyst for HER in 0.5 mol L^−1^ H_2_SO_4_ solution and 1 mol L^−1^ KOH solution and had better electrocatalytic performance in 0.5 mol/L H_2_SO_4_ solution.

Figure S6a–d shows the CV diagrams of Ti_3_C_2_T_x_, Co/Ti_3_C_2_T_x_-32, Pt/Ti_3_C_2_T_x_-6.64, and PtCo/Ti_3_C_2_T_x_-32 at different scanning rates in 1 mol L^−1^ KOH solution (Supporting Information). The C_dl_ value of the above catalyst was calculated by Equation (2), and the C_dl_ values of Ti_3_C_2_Tx, Co/Ti_3_C_2_T_x_-32, Pt/Ti_3_C_2_T_x_-6.64, and PtCo/Ti_3_C_2_T_x_-32 catalysts were shown in Figure S6e (Supporting Information). It can be seen from Figure S6e that the C_dl_ values of Ti_3_C_2_T_x_, Co/Ti_3_C_2_T_x_-32, Pt/Ti_3_C_2_T_x_-6.64, and PtCo/Ti_3_C_2_T_x_-32 were 0.03 mF cm^−2^, 0.94 mF cm^−2^, 1.83 mF cm^−2^, and 15.06 mF cm^−2^, respectively. In order to further explore the HER kinetics of these above catalysts, the corresponding Tafel slope values were obtained through the LSV curves of each catalyst, and the results were shown in Figure S6f (Supporting Information). It can be seen from Figure S6f that the Tafel slope of PtCo/Ti_3_C_2_T_x_-32 catalyst was 105.17 mV dec^−1^, which indicates that the Volmer–Heyrovsky step is rate limiting. The Tafel slopes of Pt/Ti_3_C_2_T_x_-6.64, Co/Ti_3_C_2_T_x_-32, and Ti_3_C_2_T_x_ were 98.44 mV dec^−1^, 196.52 mV dec^−1^, and 262.72 mV dec^−1^, respectively. The Tafel slope of PtCo/Ti_3_C_2_T_x_-32 was significantly reduced when it was compared with Pt/Ti_3_C_2_T_x_-6.64, Co/Ti_3_C_2_T_x_-32, and Ti_3_C_2_T_x_, indicating the kinetic process is faster and the HER activity is better.

The electro-catalytic HER performance of PtCo/Ti_3_C_2_T_x_-32 in 0.5 M H_2_SO_4_ was compared with that of the MXene-based catalyst reported in the literature [12,27,28,29,30,31,32], and the results were shown in Table 2. Furthermore, the electro-catalytic HER performance of PtCo/Ti_3_C_2_T_x_-32 in 1 M KOH was also compared with that of the MXene-based catalyst reported in the literature [32,33,34,35,36], and the results were shown in Table 3.

As can be seen from Table 2 and Table 3, PtCo/Ti_3_C_2_T_x_-32 presents lower overpotential and Tafel slope in 0.5 M H_2_SO_4_ when they were compared with the results reported by other studies. However, the results for 1 M KOH are opposite to those for 0.5 M H_2_SO_4_. Therefore, although PtCo/Ti_3_C_2_T_x_-32 can be used as an HER electrocatalyst in both acidic and alkaline electrolytes, it is more suitable as a promising HER electrocatalyst under acidic electrolyte conditions.

### 3.4. Theoretical Calculation (DFT)

**Figure 11:**
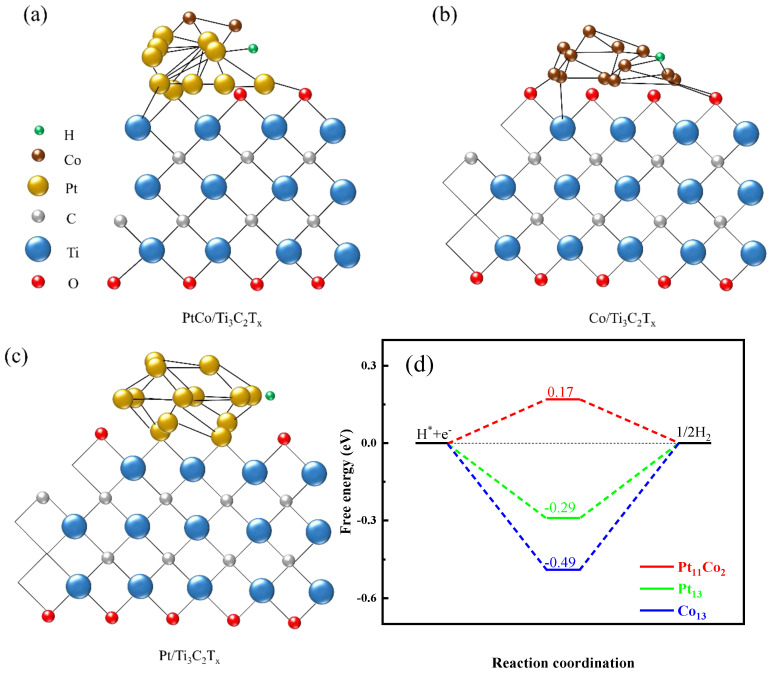

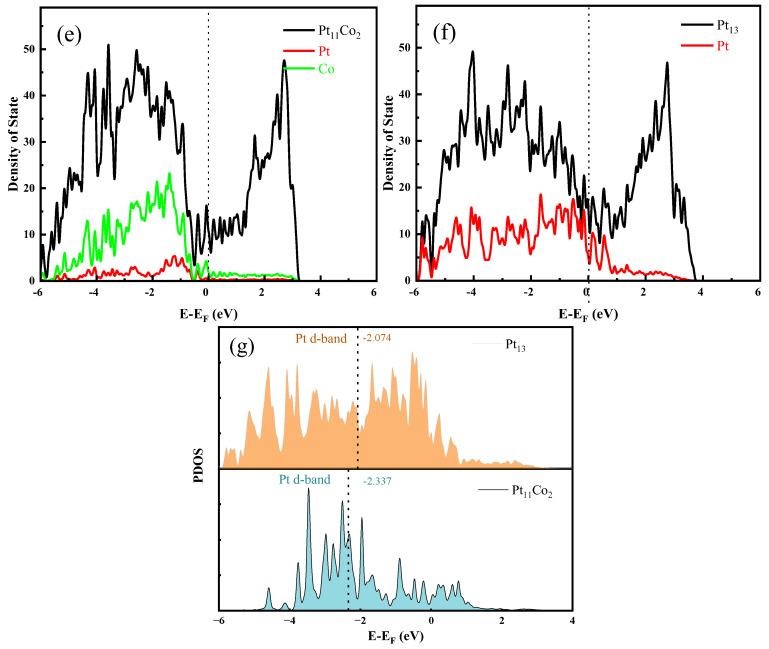
Simulation structure of (**a**): PtCo/Ti_3_C_2_T_x_, (**b**): Co/Ti_3_C_2_T_x_, (**c**): Pt/Ti_3_C_2_T_x_; (**d**): ΔG_H*_ of Co/Ti_3_C_2_T_x_, Pt/Ti_3_C_2_T_x_, and PtCo/Ti_3_C_2_T_x_; PDOS diagram of Pt atoms on the surface of, (**e**): PtCo alloy, (**f**): Pt, (**g**) d-band center diagram of Pt.

In order to further elucidate the role of Pt and Co of PtCo/Ti_3_C_2_T_x_ on electro-catalytic HER, three theoretical models (Pt_13_, Co_13_, and Pt_2_Co_11_) were established based on the types of metal clusters on Ti_3_C_2_T_x_ substrate and the relative proportions of atoms in the clusters for DFT calculation. The simulation structures of PtCo/Ti_3_C_2_T_x_, Co/Ti_3_C_2_T_x_, and Pt/Ti_3_C_2_T_x_, and calculation results were demonstrated in Figure 11a–d, respectively.

It can be seen from Figure 11d that the ΔG_H*_ of Pt/Ti_3_C_2_T_x_, Co/Ti_3_C_2_T_x_, and PtCo/Ti_3_C_2_T_x_ were −2.9 eV, −4.9 eV, and 0.17 eV, respectively. Since the ΔG_H*_ value of PtCo/Ti_3_C_2_T_x_ is closest to zero, this indicates that it has the best electro-catalytic HER performance. It is consistent with the experimental results. In order to further study the effect of Co on improving the electrocatalysis HER performance of PtCo/Ti_3_C_2_T_x_, projected state density (PDOS) information of PtCo/Ti_3_C_2_T_x_, Pt/Ti_3_C_2_T_x_, and Co/Ti_3_C_2_T_x_ catalysts was extracted, as shown in Figure 11e,f. According to the PDOS infographics of these above electrocatalysts, the d-band center of Pt atom on the surface of Pt metal and PtCo alloy was calculated and the results were shown in Figure 11g. As can be seen from Figure 11g, the d-band center of Pt/Ti_3_C_2_T_x_ and PtCo/Ti_3_C_2_T_x_ are −2.074 eV and −2.337 eV, respectively. It can be concluded that after PtCo alloying, the d-band center of Pt has an obvious negative displacement. This is due to the electronic coupling between Co and Pt, which causes the charge to be redistributed around Pt, thus moving the d-band center of Pt away from its E_f_ level. It is worth noting that the lower d-band center reduces the energy of the antibonding state, allowing more electrons to fill the antibonding state. The more valence electrons, the more of them occupy the antibonding state. It weakens the interatomic bonds in the compound, thereby reducing ΔG_H*_ [37]. As a result, low ΔG_H*_ will promote the desorption of H* from the surface of Pt, thereby increasing the electro-catalytic HER performance of PtCo/Ti_3_C_2_T_x_.

## 4. Conclusions

A series of PtCo/Ti_3_C_2_T_x_-Y (Y: 16, 32, and 320) electrocatalysts were prepared from the anchoring of PtCo alloy by Ti_3_C_2_T_x_ nanosheets. The PtCo/Ti_3_C_2_T_x_-32 exhibited the best HER performance with a low overpotential of 36 mV to reach a current density of 10 mA cm^−2^ and a small Tafel slope of 66.37 mV dec^−1^ in 0.5 mol L^−1^ H_2_SO_4_ solution. The smaller Tafel slope value indicates that the PtCo/Ti_3_C_2_T_x_-32 catalyst has a fast kinetic process and follows the Volmer–Heyrovsky mechanism in 0.5 mol L^−1^ H_2_SO_4_ solution. DFT calculation shows that the PtCo alloy makes the PtCo/Ti_3_C_2_T_x_ catalyst have a suitable d-band center and ΔG_H*_ value close to 0, which greatly promotes desorption of H* from the surface of Pt, thereby increasing HER activity. PtCo/Ti_3_C_2_T_x_ could be used as an efficient and stable HER electrocatalyst and had a broad application prospect.

## Figures and Tables

**Table 1:** Specific surface area, pore volume, and average pore diameter of each catalyst.

Catalyst	Specific Surface Area (m^2^/g)	Pore Volume (cm^3^/g)	Pore Diameter (nm)
Ti_3_C_2_T_x_	2.96	0.0075	10.77
Co/Ti_3_C_2_T_x_-32	15.41	0.015	38.84
Pt/Ti_3_C_2_T_x_-6.64	16.37	0.028	6.75
PtCo/Ti_3_C_2_T_x_-32	59.31	0.039	2.66

**Table 2:** Comparison of electro-catalytic HER performance of the MXene-based catalysts in 0.5 M H_2_SO_4_ to reach a current density of 10 mA cm^−2^.

Type of Electrocatalyst	Electro-Catalytic HER Performance		Refs.
	Overpotential (mV)	Tafel Slope (mV dec^−1^)	
Pt/Ti_3_C_2_T_x_	34	29.7	[12]
Ni SA@Ti_3_C_2_T_x_	63	70	[27]
Ti_3_C_2_T_x_/Pt	180	48	[28]
P-Ti_3_C_2_T_x_@NiCoP	115	76	[29]
MoSe_2_/O@Ti_3_C_2_T_x_	121	82	[30]
Ru@Ti_3_C_2_T_x_-NS	46.75	30.6	[31]
RuCo-Ti_3_C_2_T_x_	60	34.8	[32]
PtCo/Ti_3_C_2_T_x_-32	36	66.37	This work

**Table 3:** Comparison of electro-catalytic HER performance of the MXene-based catalysts in 1 M KOH to reach a current density of 10 mA cm^−2^.

Type of Electrocatalyst	Electro-Catalytic HER Performance		Refs.
	Overpotential (mV)	Tafel Slope (mV dec^−1^)	
RuCo-Ti_3_C_2_T_x_	52	38.7	[32]
V2C MXene/ZIF-67	197	76	[33]
PtNi@PS-TCT (phosphorus and sulfur-functionalized and self-adapting Ti^3+^ species defect decorated Ti_3-x_C_2_T_y_ MXene)	56.1	81	[34]
Mn-CoP/MXene	95	54.1	[35]
V2CTx/Ag-NPs	32	114	[36]
PtCo/Ti_3_C_2_T_x_-32	101	105.17	This work

## Data Availability

Data are contained within the article and Supplementary Materials.

## Supplementary Materials

The following supporting information can be downloaded at: https://www.mdpi.com/article/10.3390/molecules29235793/s1, Figure S1: (a) SEM image of PtCo/Ti_3_C_2_T_x_-32, (b) EDS mapping of PtCo/Ti_3_C_2_T_x_-32, (c) Pt element, (d) Co element, (e) C element, (f) Ti element; (g) element proportion at each point in map; Figure S2: XPS diagram of Ti_3_C_2_T_x_; Figure S3: XPS diagram of Co/Ti_3_C_2_T_x_-32; Figure S4: XPS diagram of Pt/Ti_3_C_2_T_x_-6.64; Figure S5: Contact angles of (a) Ti_3_C_2_T_x_, (b) Pt/Ti_3_C_2_T_x_-6.64, (c) Co/Ti_3_C_2_T_x_-32 and (d) PtCo/Ti_3_C_2_T_x_-32; Figure S6: CV diagram of (a) Ti_3_C_2_T_x_, (b) Co/Ti_3_C_2_T_x_-32, (c) Pt/Ti_3_C_2_T_x_-6.64, (d) PtCo/Ti_3_C_2_T_x_-32; (e) C_dl_ values and (f) Tafel slopes of Ti_3_C_2_T_x_, Co/Ti_3_C_2_T_x_-32, Pt/Ti_3_C_2_T_x_-6.64 and PtCo/Ti_3_C_2_T_x_-32 in 1 mol L^−1^ KOH solution.
