# Supplementary material for: MXene Anchored with Platinum Cobalt Alloy as an Efficient and Stable Electrocatalyst for Hydrogen Evolution
Source: Molecules. 2024 Dec 7;29(23):5793. doi: 10.3390/molecules29235793 (PMC11642977; doi:10.3390/molecules29235793)
Supplement: Supplementary file 1 [file molecules-29-05793-s001.zip › molecules-3309287-supplementary.pdf]

Supporting information

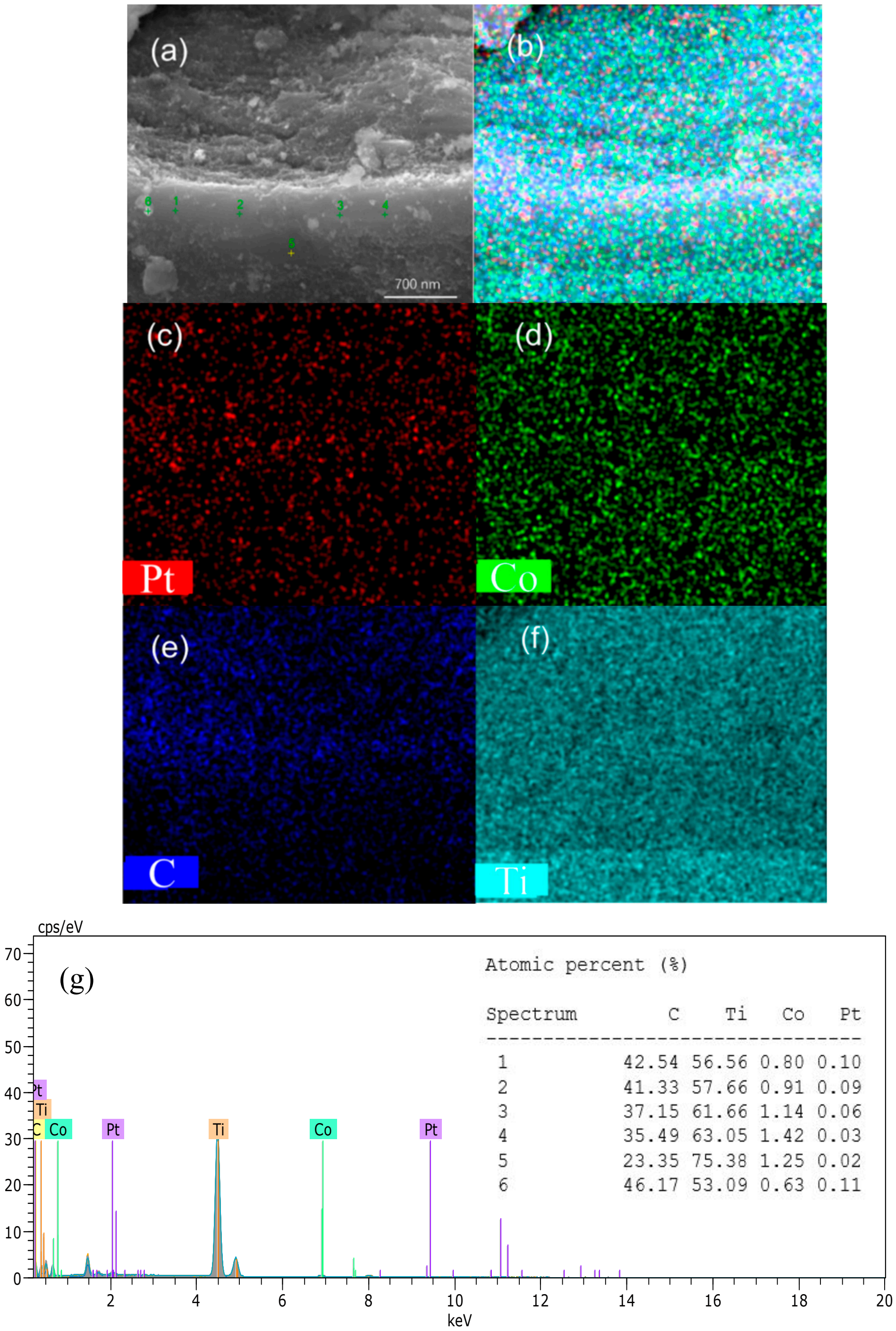

Fig.S1.(a) SEM image of PtCo/$Ti_3C_2T_x$-32, (b) EDS mapping of PtCo/$Ti_3C_2T_x$-32, (c) Pt element, (d) Co element, (e) C element, (f) Ti element; (g) element proportion at each point in map

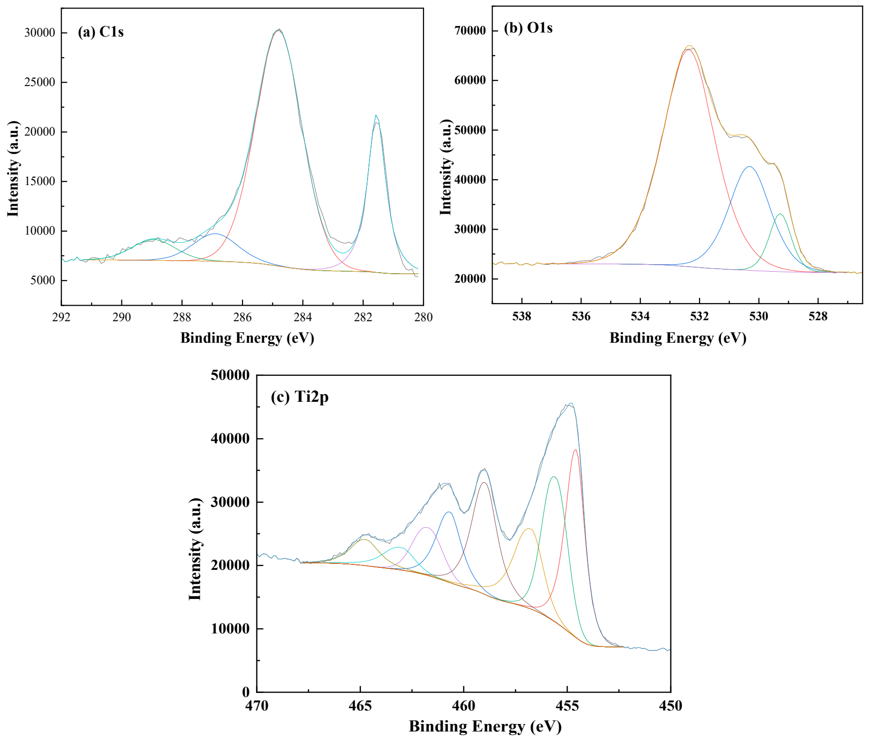

Fig.S2 XPS diagram of $Ti_3C_2T_x$

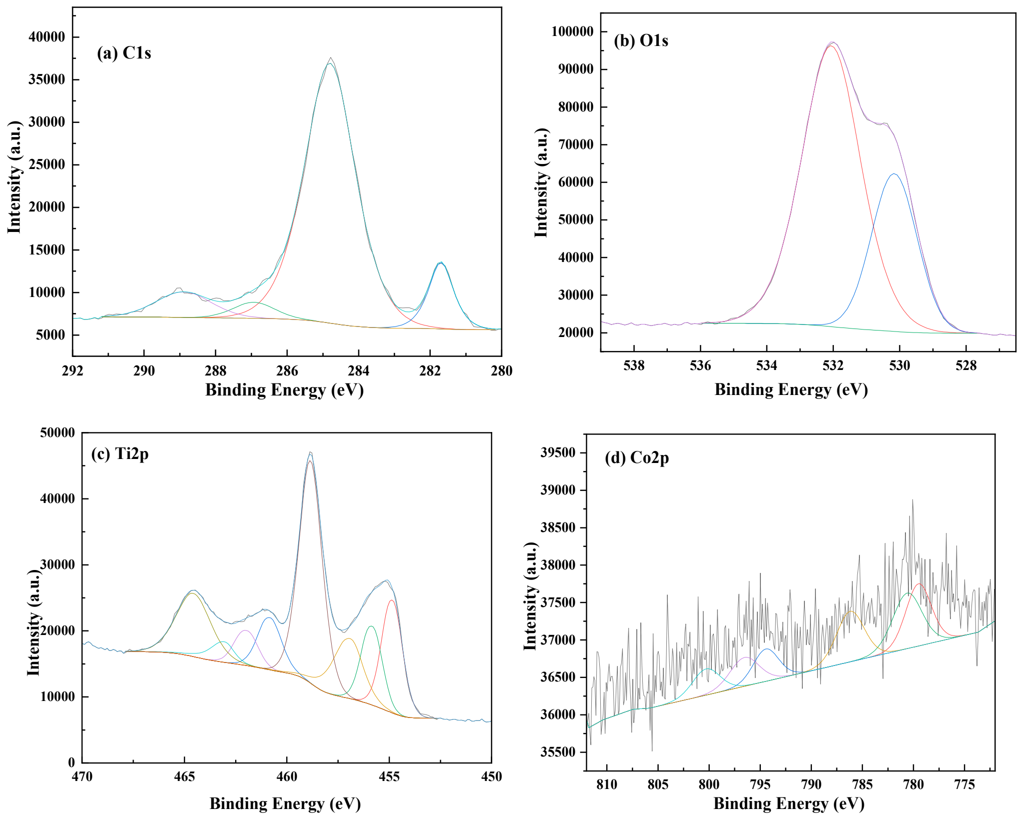

Fig.S3 XPS diagram of Co/$Ti_3C_2T_x$-32

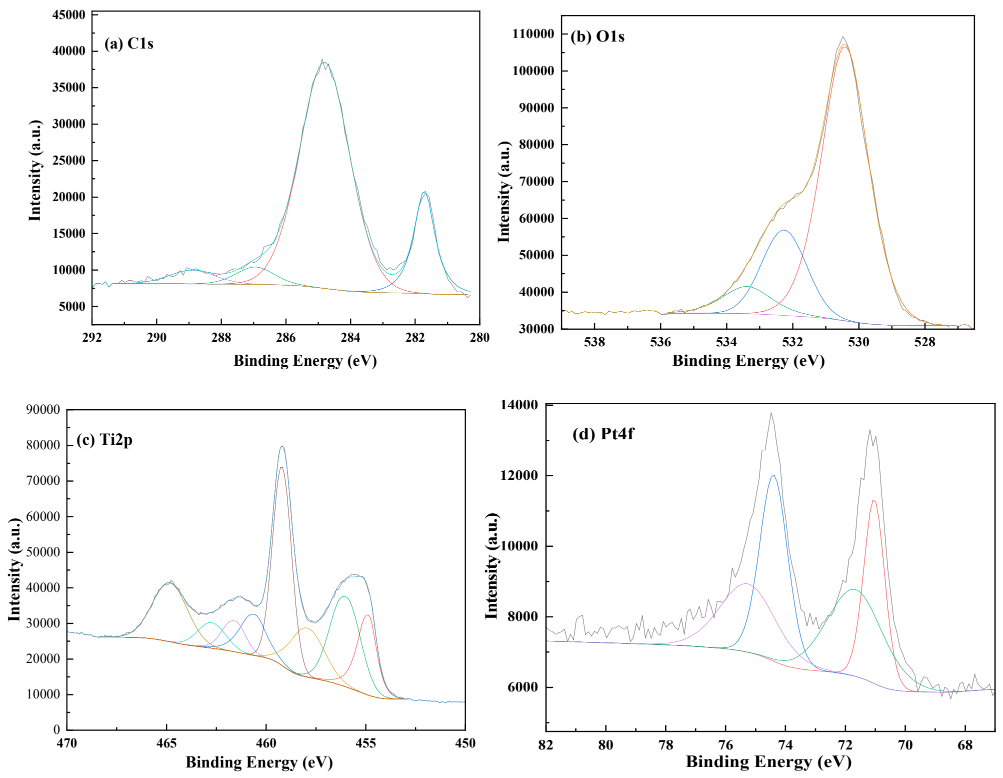

Fig.S4 XPS diagram of $Pt/Ti_3C_2T_x$-6.64

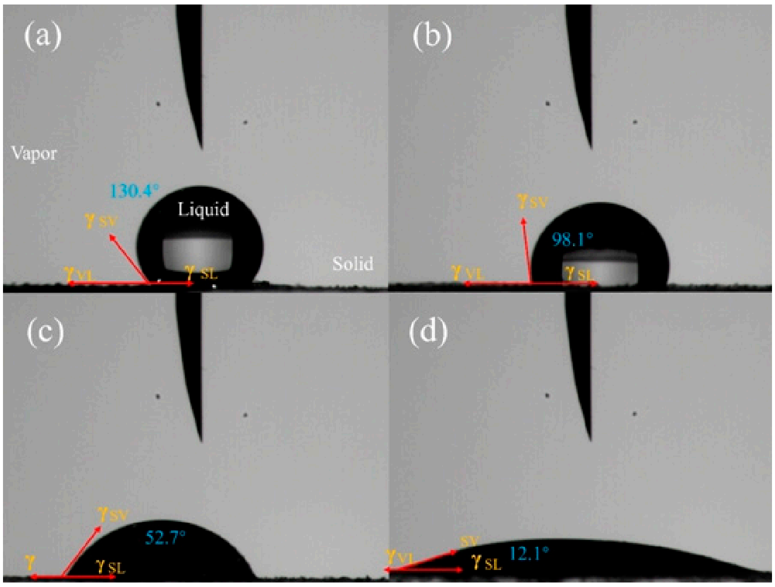

Fig.S5 Contact angles of (a) $Ti_3C_2T_x$，(b) $Pt/Ti_3C_2T_x$-6.64，(c) $Co/Ti_3C_2T_x$-32 and (d) $PtCo/Ti_3C_2T_x$-32

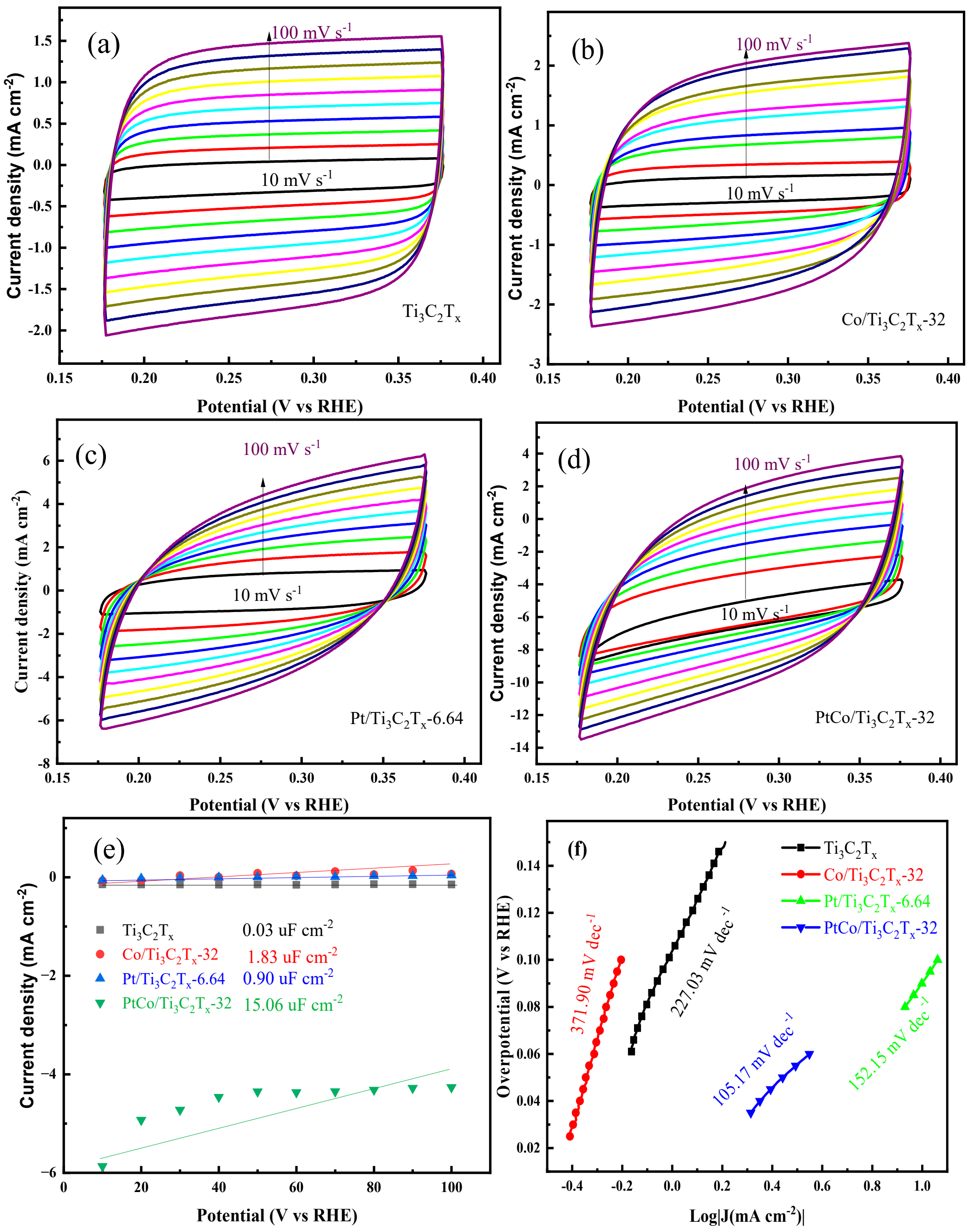

Fig.S6 CV diagram of (a) $Ti_3C_2T_x$, (b) Co/$Ti_3C_2T_x$-32, (c) Pt/$Ti_3C_2T_x$-6.64, (d) PtCo/$Ti_3C_2T_x$-32; (e) C_{dl} values and (f) Tafel slopes of $Ti_3C_2T_x$, Co/$Ti_3C_2T_x$-32, Pt/$Ti_3C_2T_x$-6.64 and PtCo/$Ti_3C_2T_x$-32 in 1 mol L^{-1} KOH solution.
